# Complement Activation as a Predictor of Postoperative Delirium in Elderly Spine Surgery Patients

**DOI:** 10.3390/ijms27021077

**Published:** 2026-01-21

**Authors:** Antje Vogelgesang, Hannah Wolf, Sarah Strack, Agnes Flöel, Henry W. S. Schroeder, Jonas Müller, Jan-Uwe Müller, Angelika Fleischmann, Robert Fleischmann, Diana Pauly, Johanna Ruhnau

**Affiliations:** 1 Department of Neurology, University Medicine Greifswald, 17475 Greifswald, Germany; 2 Experimental Ophthalmology, University Marburg, 35043 Marburg, Germany; 3 Department of Neurosurgery, University Medicine Greifswald, 17475 Greifswald, Germany

**Keywords:** biomarker, complement activation, delirium, postoperative cognitive dysfunction, spine surgery

## Abstract

Postoperative delirium (POD) is a frequent and serious complication among elderly surgical patients. Despite its clinical relevance, reliable biomarkers for early identification and pathophysiological insight remain limited. Recent evidence implicates systemic immune activation and complements dysregulation as contributors to cognitive decline after surgery. This study investigated the association between perioperative levels of selected complement pathway proteins and both the incidence and severity of POD. Methods: We performed a secondary analysis of 22 patients aged ≥ 60 years from the prospective CONFESS cohort undergoing elective spine surgery. Complement proteins (C1q, C2, C4), mannose-binding lectin (MBL), Factor D [FD], Factor B [FB], Factor I [FI] were quantified from blood samples collected at baseline, preoperatively, and on postoperative days 1 and 2. POD was assessed using the Nursing Delirium Screening Scale (Nu-DESC) and Diagnostic and Statistical Manual of Mental Disorders, Fifth Edition criteria. Delirium severity was rated with the Confusion Assessment Method–Severity (CAM-S) scale. Associations were tested using univariate and multivariate regression analyses. Preoperative levels of FD and C2 were significantly elevated in patients who developed POD (FD: *p* = 0.023; C2: *p* = 0.044), while C4 levels trended lower. FD remained an independent predictor of POD in multivariate regression (*p* = 0.049), although cognitive performance was the only significant predictor when adjusted for surgery duration. Delirium severity was associated with perioperative reductions in C1q, FI, and FB and with increased MBL levels, explaining up to 43% of CAM-S score variance. These findings highlight the role of complement activation—particularly FD, C2, MBL—in the development and clinical expression of POD. Complement profiling may offer a novel approach for risk stratification and therapeutic targeting in perioperative neurocognitive disorders.

## 1. Introduction

Postoperative neurocognitive disorders (PNDs), encompassing postoperative delirium (POD), delayed neurocognitive recovery, and postoperative cognitive dysfunction (POCD), are among the most frequent and debilitating complications in older surgical patients. POD is characterized by an acute, fluctuating disturbance in attention and cognition, typically occurring within the first 72 h after surgery, and affects up to 50% of older adults depending on surgical type and vulnerability profile [1,2,3]. In contrast, POCD represents a more subtle and insidious decline in memory, attention, and executive function that may persist for weeks to months, with prevalence rates of approximately 10–15% at three months, declining to only about 1% one year after surgery [4,5]. These cognitive impairments are clinically relevant as they are associated with prolonged hospitalization, impaired independence, reduced social functioning, increased institutionalization, and higher mortality rates [6,7,8]. Importantly, even middle-aged patients (40–60 years) can be affected, although with lower frequency than in the elderly [9].

The etiology of PNDs is multifactorial, but accumulating evidence points to neuroinflammation as a central mechanism linking systemic surgical insult with central nervous system (CNS) dysfunction [10,11,12]. Perioperative immune activation releases cytokines that disrupt the blood–brain barrier, activate microglia, and injure neurons. Elevated Interleukin-6, C-reactive protein, and S100*β* have been consistently associated with POD and POCD [13], and Interleukin-1*β* inhibition prevents postoperative cognitive decline in aged rodents [10]. More recently, biomarkers such as soluble neurofilament light chain, soluble triggering receptor expressed on myeloid cells 2 (sTREM2), and Gasdermin D have emerged as predictors of cognitive impairment [13,14]. Increased perioperative soluble triggering receptor expressed on myeloid cells 2 (sTREM2) and Gasdermin D correlate with memory deficits, while higher soluble neurofilament light chain indicates brain atrophy and poorer cognitive trajectories [15]. Together, these findings highlight the interplay of systemic inflammation and neurodegeneration in postoperative cognitive outcomes.

In this context of neuroinflammation, the complement system represents a critical yet underexplored pathway. As an integral component of innate immunity, the complement cascade contributes to host defense, clearance of apoptotic cells, and modulation of adaptive immunity. It can be activated via the classical, lectin, or alternative pathways, which converge on the central components C3 and C5, promoting opsonization, inflammation, and cell lysis [16]. While tightly regulated under physiological conditions, complement dysregulation has been implicated in neurodegenerative diseases such as Alzheimer’s disease, where excessive activity fosters synaptic pruning, neuronal loss, and progressive cognitive decline [17,18]. Emerging perioperative studies suggest that similar mechanisms may operate in PNDs. Elevated preoperative levels of Factor D (FD) and Complement factor 2 (C2) have been linked to an increased risk of POD, while reduced preoperative levels of the C4B isoform were observed in affected individuals. Moreover, delirium severity has been associated with altered concentrations of complement components such as Complement C1r (C1R), Factor I (FI), Factor B (FB), and Mannose-Binding Lectin (MBL), indicating a dynamic regulation of the complement system during the perioperative period [19]. Of additional interest are the Factor H-related proteins (FHRs), structurally similar to Factor H but thought to enhance complement activation by competing for binding sites [20]. Although FHRs have been linked to autoimmune diseases and severe infections, their role in perioperative neurocognitive decline or postoperative delirium remains underexplored.

Based on this evidence, we hypothesized that perioperative alterations in specific complement pathway proteins—including C1q, C2, C4, Factor D (FD), FB, FI, and MBL—are associated with POD incidence and severity. By integrating complement analysis into established biomarker frameworks, this study aims to refine our understanding of the immunological drivers of postoperative neurocognitive disorders and to identify potential biomarkers for risk stratification and therapeutic targeting.

## 2. Results

### 2.1. Results–Baseline and Cognitive Characteristics

Baseline characteristics were largely comparable between patients with and without postoperative delirium (non-delirium vs. delirum) (Table 1). Age, sex distribution, and years of education did not differ significantly between groups (all *p* > 0.10). Patients who developed delirium, however, underwent significantly longer surgical procedures compared with non-delirious patients (245 ± 132.7 min vs. 168.9 ± 83.0 min, *p* = 0.021).

Cognitive performance changes are summarized in Table 1. No statistically significant group differences were observed across any of the assessed neuropsychological domains.

### 2.2. Delirium Incidence

Preoperative levels of Factor D (FD-V0) and C2 (C2-V0) were higher in patients who developed POD than in those who did not (FD: 9.73 ± 6.25 pg/mL vs. 6.11 ± 2.81 pg/mL; *p* = 0.023; C2: 5.32 ± 4.30 pg/mL vs. 3.25 ± 1.46 pg/mL; *p* = 0.044). C4-V0 levels were lower in patients who developed POD compared with those who did not (2.58 ± 1.16 pg/mL vs. 2.75 ± 1.60 pg/mL), although this difference did not reach statistical significance (*p* = 0.48). In univariate logistic regression analysis, higher baseline FD-V0 levels were significantly associated with an increased risk of POD (OR = 1.221; 95% CI: 1.001−1.490; *p* = 0.049) (Table 2).

Multivariable analysis excluding preoperative cognitive performance and surgery time identified FD-V0 as an independent predictor (*p* = 0.049) for POD. When adjusted for CERAD-NP and surgery time, preoperative cognitive performance was the only variable that remained significant (*p* = 0.011; Table 3).

All other biomarker concentrations did not have a significant effect at any of the measurement time points.

### 2.3. Delirium Severity

Delirium severity was assessed using the Confusion Assessment Method–Severity Score (CAM-S), derived from CAM items. Fifteen patients developed delirium. Among delirious patients, the median CAM-S score was 4.0 (IQR 0–8), with values ranging from 0 to 14, indicating substantial variability in delirium severity. According to established CAM-S short-form cut-offs, 47% of patients had mild, 7% moderate, and 47% severe delirium. CAM-S scores were zero in patients without delirium.

Linear regression analyses were performed to examine the association between complement biomarker concentrations and delirium severity, assessed using the Confusion Assessment Method–Severity (CAM-S) score. CAM-S severity was analyzed as a continuous dependent variable, and univariate linear regression models were calculated for biomarker concentrations measured at baseline (V0) and at postoperative time points (V1, V2.1, and V2.2).

**Figure 1:**
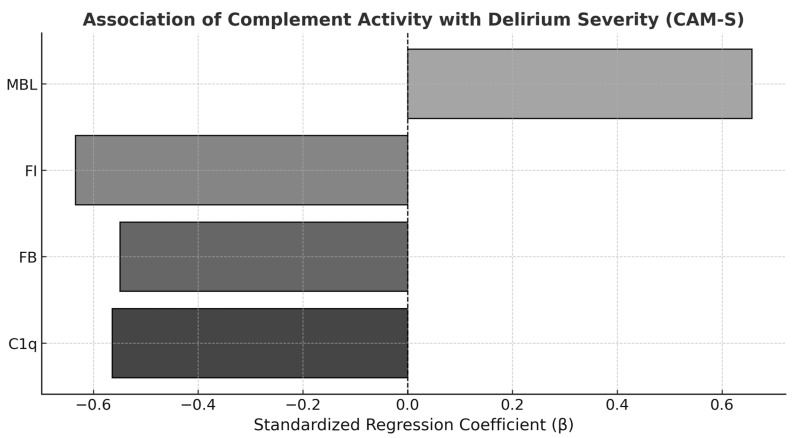
**Association of complement activity with delirium severity.** Univariate linear regression analyses demonstrated significant associations between perioperative complement activity and delirium severity as assessed by the Confusion Assessment Method–Severity score (CAM-S). Lower perioperative C1q levels (V1) were associated with higher delirium severity (β = −0.564, *p* = 0.036, R^2^ = 0.32), as were reduced factor I (FI) levels on postoperative day 1 (V1.2) (β = −0.634, *p* = 0.020, R^2^ = 0.40) and decreased factor B (FB) levels at V2.2 (β = −0.549, *p* = 0.052, R^2^ = 0.30). In contrast, higher mannan-binding lectin (MBL) levels on postoperative day 1 were positively associated with CAM-S scores (β = +0.657, *p* = 0.015, R^2^ = 0.43). Various grey tones were used to differentiate the complement factors.

Lower intraoperative C1q concentrations (V1) were significantly associated with higher CAM-S scores (β = −0.564, *p* = 0.036), explaining 31.8% of the variance in delirium severity (R^2^ = 0.318). Similarly, reduced Factor I (FI) levels on postoperative day 1 (V2.1) were significantly associated with increased delirium severity (β = −0.634, *p* = 0.020; R^2^ = 0.402). Factor B (FB) levels on postoperative day 2 (V2.2) showed a trend toward significance, with lower concentrations associated with higher CAM-S scores (β = −0.549, *p* = 0.052; R^2^ = 0.302). In contrast, higher Mannose-Binding Lectin (MBL) concentrations on postoperative day 1 (V2.1) were significantly associated with greater delirium severity (β = 0.657, *p* = 0.015), accounting for 43.1% of the variance (R^2^ = 0.431). All other complement biomarkers did not show a significant association with delirium severity at any assessed time point (Figure 1).

## 3. Discussion

This study examined the role of complement pathway proteins in perioperative neurocognitive disorders, particularly postoperative delirium and its severity. By integrating complement biomarkers into established inflammation-based models, our findings extend current understanding of the immunological mechanisms underlying perioperative neurocognitive disorders.

In this study, we observed associations between specific components of the complement system and both the occurrence and severity of POD in elderly patients undergoing spine surgery. Elevated preoperative levels of FD and C2 were associated with an increased risk of POD, while lower C4 levels showed a trend toward POD development. Together, these findings suggest that perioperative complement activity may be linked to susceptibility to postoperative neurocognitive dysfunction.

The decrease in C4 levels observed in patients who developed POD may reflect a dynamic inflammatory response rather than impaired immune capacity. Previous proteomic studies have demonstrated time-dependent changes in complement-related proteins during delirium, supporting the notion that reduced circulating C4 could be indicative of increased activation and consumption of the classical complement pathway during perioperative inflammatory stress [21]. In this context, elevated preoperative FD levels may indicate a primed alternative complement pathway and heightened innate immune responsiveness and were associated with a modestly increased risk of postoperative delirium (OR 1.22, 95% CI 1.00–1.49), potentially contributing to increased vulnerability to perioperative neuroinflammatory signaling rather than acting as a strong independent risk factor.

In addition to POD incidence, we found that complement dysregulation was also associated with delirium severity. Lower levels of C1q, FI, and FB, as well as higher levels of MBL, were correlated with increased delirium severity. These findings suggest that complement activity may not only be involved in the development of POD but could also influence its clinical course, pointing toward pathway-specific regulation of the complement system during the perioperative period.

Our results are consistent with and extend previous evidence implicating the complement cascade in neuroinflammatory processes. Dysregulated complement activity has been widely described in chronic neurodegenerative disorders, particularly Alzheimer’s disease [17]. The present study suggests that similar mechanisms may also be relevant in the setting of acute postoperative neurocognitive dysfunction. Unlike prior studies that primarily focused on chronic neurodegeneration or exploratory proteomic analyses [17,18,19], our work applied a targeted assessment of multiple complement pathway components in a defined perioperative context. This approach allows pathway-level interpretation and links complement alterations not only to POD occurrence but also to delirium severity.

The observed associations with FD and C2 are of particular interest, as these proteins represent initiating components of the alternative and classical complement pathways, respectively. Their elevation may reflect a pre-existing systemic immune state that increases vulnerability to postoperative central nervous system insults, although causal relationships cannot be inferred from the present data. Similarly, the positive association between MBL levels and delirium severity suggests a potential contribution of lectin pathway activation to acute neuroinflammatory responses, in line with previous reports implicating MBL in inflammatory injury following ischemia or infection.

From a clinical perspective, these findings suggest that complement proteins may have potential value as biomarkers for POD risk stratification. Early identification of patients at increased risk based on complement profiling could, in principle, support preventive strategies such as optimized anesthetic management, enhanced postoperative surveillance, or targeted immunomodulatory approaches. Furthermore, the complement system may represent a potential therapeutic target. Inhibition of specific complement components, including C3, has shown beneficial effects in preclinical models of neuroinflammation and neurodegeneration and may warrant further investigation in the context of postoperative delirium [22,23,24].

Mechanistically, perioperative complement dysregulation may contribute to POD through amplification of neuroinflammatory signaling and increased synaptic vulnerability. Complement components such as C1q and downstream effectors are known to participate in microglial activation and complement-mediated synaptic pruning—processes that are essential during development but may become detrimental when aberrantly reactivated in the aging brain [25,26]. Surgical stress and systemic inflammation may further impair blood–brain barrier integrity, facilitating interactions between peripheral immune mediators and central complement pathways. In this setting, reduced circulating levels of C1q and regulatory factors could reflect increased local consumption or deposition within the central nervous system, potentially promoting microglial activation and synaptic dysfunction that manifest clinically as delirium [27]. While these mechanistic considerations remain speculative, they provide a biologically plausible framework linking peripheral complement alterations to postoperative neurocognitive disturbances and underscore the need for future studies integrating longitudinal biomarker assessments with neuroimaging and experimental approaches.

Several limitations should be considered when interpreting the results of this study. The sample size of the biomarker analysis was modest, which may have limited statistical power and the ability to account for all potentially relevant confounders. The analyses were conducted within an exploratory framework, and no formal correction for multiple comparisons was applied; therefore, the findings should be interpreted with appropriate caution. While associations between complement activation and postoperative delirium were observed, causal conclusions cannot be drawn. Complement activation may reflect, at least in part, broader perioperative inflammatory processes. In addition, the influence of perioperative factors such as anesthesia techniques and preoperative medication use cannot be completely ruled out. Future studies with larger cohorts are warranted to confirm these findings and to further investigate the underlying biological mechanisms.

In summary, our data provide novel evidence that dysregulation of classical, alternative, and lectin complement pathways is linked to both the incidence and severity of postoperative delirium. These findings emphasize the central role of complement in perioperative neuroinflammation and highlight its potential as a target for biomarker development and therapeutic intervention.

## 4. Materials and Methods

### 4.1. Study Design and Participants

This secondary analysis is based on data from the Cognitive Dysfunction Following Elective Spine Surgery in Elderly Patients (CONFESS) study, a prospective, single-center observational trial conducted at the University Medicine Greifswald, Germany. The study was approved by the institutional ethics board (BB 192/17) and registered with ClinicalTrials.gov (NCT03486288). All participants provided written informed consent. Eligible participants were adults aged ≥60 years undergoing elective spine surgery between February 2018 and March 2020. Exclusion criteria included pre-existing dementia, psychiatric illness, substance abuse, or urgent surgery. Of 99 enrolled patients, 22 with complete biomarker and cognitive data were included in this biomarker analysis.

### 4.2. Study Protocol and Assessments

Clinical and cognitive assessments were performed at four defined time points. At baseline (V0), patients underwent CERAD-NP cognitive testing together with frailty index evaluation, comorbidity scoring, and assessment of activities of daily living (ADL). Perioperatively (V1), blood samples were collected and surgical risk was evaluated. On postoperative days 1 and 2 (V2.1 and V2.2), delirium was assessed using the Nursing Delirium Screening Scale (Nu-DESC) and Diagnostic and Statistical Manual of Mental Disorders, Fifth Edition (DSM-5) criteria, and additional blood samples were obtained. Participants were categorized into two groups based on the occurrence of postoperative delirium. Postoperative delirium was assessed using standardized diagnostic criteria, and patients were classified as either delirium-positive or delirium-negative according to the presence or absence of delirium during the postoperative observation period. Three months after surgery (V3), cognitive function was re-evaluated using the CERAD-NP test battery. The primary outcome was the change in cognitive performance, calculated as ΔCERAD (V3–V0). Delirium severity was quantified using the CAM-S score. Educational attainment was recorded as the highest completed educational level and coded as an ordinal variable with increasing values indicating higher education. In addition, years of formal education were recorded as a continuous variable and used for descriptive analyses.

### 4.3. Complement Biomarker Measurement

Blood samples were collected and analyzed at V0, V1, V2.1, and V2.2. For complement system analyses, serum samples were used exclusively, stored at −80 °C, and handled under standardized conditions. Repeated freeze–thaw cycles were avoided, as these are known to affect the stability and functional integrity of complement proteins. Sample stability under these storage conditions was not independently validated in the present study; however, the stability of serum complement proteins at −80 °C has been reported in previous studies [28,29]. All samples were processed and stored uniformly, ensuring comparability across study groups. Analytes included classical (C1, C2, C4), alternative (FD, FB), mannose-binding lectin (MBL), and regulatory (FI) pathway components. For analysis and quantification of complement components, the MILLIPLEX^®^ Human Complement panels 1 and 2 (Merck, Darmstadt, Germany) were used. Samples were measured twice as median fluorescence intensity (MFI) values in a Bio-Plex 200 (Bio-Rad Laboratories, Inc., Hercules, CA, USA). Serum samples were diluted 1:1000 for Panel 1 and 1:40,000 for Panel 2 according to manufactures instruction.

### 4.4. Statistical Analysis

Study data were transferred from paper-based case report forms into an electronic data capture system (CentraXX, Kairos GmbH, Bochum, Germany), which enabled real-time verification of data completeness, plausibility, and value ranges. Statistical analyses were performed using IBM SPSS Statistics version 28 (IBM Corp., Armonk, NY, USA).

Continuous variables are presented as mean ± standard deviation (SD). All laboratory measurements were performed in duplicate, and the mean of both measurements was used for all analyses. Normality of continuous variables was assessed using the Shapiro–Wilk test and visual inspection of Q–Q plots. Homogeneity of variance was evaluated where applicable using standard diagnostic procedures within SPSS. Group comparisons of continuous variables were performed using parametric or non-parametric methods as appropriate.

To evaluate associations between preoperative complement protein levels and the incidence of postoperative delirium (POD), univariate binary logistic regression analyses were performed. Results are reported as odds ratios (ORs) with 95% confidence intervals (CIs). Complement biomarkers showing significant associations or trends in univariate analyses were subsequently included in multivariable logistic regression models. Multivariable models were constructed using a forward likelihood ratio approach. Additional models were adjusted for preoperative cognitive performance (CERAD-NP total score) and duration of surgery to account for potential confounding. Delirium severity was assessed using the Confusion Assessment Method–Severity Score (CAM-S), derived from CAM items. CAM-S scores were analyzed as a continuous dependent variable. Associations between complement biomarker concentrations and delirium severity were examined using univariate linear regression analyses for each biomarker at baseline (V0) and at postoperative time points (V1, V2.1, and V2.2). Standardized regression coefficients (β) and coefficients of determination (R^2^) were reported to quantify effect size and explained variance.

*p*-values < 0.05 were considered statistically significant. Biomarkers not reaching statistical significance at any time point are reported as non-significant.

## 5. Conclusions

This study suggests that complement dysregulation is associated with both the incidence and severity of postoperative delirium in elderly patients undergoing spine surgery. Elevated preoperative levels of Factor D and C2, along with postoperative alterations in C1q, FI, FB, and MBL, point toward a potential involvement of complement activation in perioperative neuroinflammatory processes. Complement proteins may therefore have value as biomarkers of cognitive vulnerability and for improving perioperative risk stratification. Larger, prospective studies are required to confirm these findings and to determine whether modulation of complement activity may help reduce postoperative neurocognitive disorders.

## Figures and Tables

**Table 1:** **Baseline characteristics of patients with and without postoperative delirium.** Values are presented as mean ± standard deviation unless otherwise indicated. *p*-values are derived from independent samples t-tests (two-sided). *CERAD:* Consortium to Establish a Registry for Alzheimer’s Disease-PLUS, SD: standard deviation.

	Non-Delirium (*n* = 22)		Delirium (*n* = 15)		
Variable	Mean	Standard Deviation	Mean (*n* = 15)	Standard Deviation	*p*-Value
Age. years (mean ± SD)	76.18	4.62	76.47	3.87	0.42
Duration of surgery. min (mean ± SD)	168.9	82.96	245	132.7	0.021
Gender. male proportion	0.55	0.51	0.6	0.51	0.38
Education. years	9.18	1.59	8.6	1.35	0.13
Education type	2.95	1.08	2.23	1.17	0.42
**CERAD Items**					
Verbal fluency	0.13	0.96	0.23	0.60	0.835
Boston Naming Test	0.22	0.66	0.39	0.99	0.640
Mini Mental State Examination	0.96	1.98	−0.68	1.42	0.083
Word list learning	0.24	2.04	0.69	1.22	0.624
Word list recall	0.17	1.03	0.16	1.19	0.980
Word list recognition	0.78	1.31	0.95	1.13	0.786
Visuoconstruction copy	0.50	2.97	0.14	2.98	0.805
Visuoconstruction recall	0.67	1.50	0.76	3.01	0.937
Trail Making Test A	−0.13	0.96	−0.05	1.05	0.877
Trail Making Test B	0.09	0.66	0.46	0.75	0.282
Phonemic fluency	−0.21	0.79	−0.01	0.82	0.583
Total score (mean)	0.29	0.70	0.28	0.75	0.969

**Table 2:** **Preoperative levels of Factor D (FD-V0); C2 (C2-V0) and C4-V0:** Univariate regression analysis, V0 = baseline; Rk.B: regression coefficient B; W: Wald statistics, OR: odds ratio, * = significant at the 0.05 level; CI: confidence intervals.

	Rk.B	W (df)	*p*	OR	CI (Lower)	CI (Upper)
FD-V0	0.200	3.862	0.049 *	1.221	1.001	1.490
C4-V0	−0.930	3.402	0.065	0.395	0.147	1.060
C2-V0	0.266	2.988	0.084	1.305	0.965	1.764

**Table 3:** **Multivariable regression analyses of FD as a predictors of postoperative delirium:** Multivariate regression analysis unadjusted and adjusted for CERAD-NP preoperative and surgery time V0 = baseline; Rk.B: regression coefficient B; W: Wald statistics, OR: odds ratio, * = significant at the 0.05 level; CI: confidence intervals.

Unadjusted	Rk.B	W (df)	*p*	OR	CI (Lower)	CI (Upper)
FD-V0	0.200	3.862	0.049 *	1.221	1.001	1.490
**Adjusted for**	**Rk.B**	**W (df)**	***p***	**OR**	**CI (lower)**	**CI (upper)**
FD-V0	0.184	1.260	0.262	1.201	0.872	1.655
surgery time	0.005	1.348	0.246	1.005	0.997	1.013
preoperative CERAD-NP	−1.952	6.391	0.011 *	0.142	0.031	0.645

## Data Availability

Data are available by the corresponding author upon reasonable request. Unrestricted publication of datasets is not covered by local directives of the General Data Protection Regulation (EU) 2016/679.

## Abbreviations

POD	Postoperative Delirium
POCD	Postoperative Cognitive Dysfunction
PND	Perioperative Neurocognitive Disorders
CAM-S	Confusion Assessment Method–Severity
CERAD-NP	Consortium to Establish a Registry for Alzheimer’s Disease–Neuropsychological Battery
Nu-DESC	Nursing Delirium Screening Scale
DSM-5	Diagnostic and Statistical Manual of Mental Disorders, Fifth Edition
CI	Cognitive Impairment / Cognitive Index (context-dependent)
MMSE	Mini-Mental State Examination
FD	Factor D
FB	Factor B
FI	Factor I
C1q, C2, C3, C4	Complement Components 1q, 2, 3, and 4
MBL	Mannose-Binding Lectin
FH	Factor H
FHR	Factor H-Related Proteins
CONFESS	Cognitive and Functional Evaluation Study in Spine Surgery
CNS	Central Nervous System
